# Human papillomavirus detection in moroccan patients with nasopharyngeal carcinoma

**DOI:** 10.1186/1750-9378-6-3

**Published:** 2011-02-25

**Authors:** Nadia Laantri, Mohammed Attaleb, Mostafa Kandil, Fadwa Naji, Tarik Mouttaki, R'kia Dardari, Khalid Belghmi, Nadia Benchakroun, Mohammed El Mzibri, Meriem Khyatti

**Affiliations:** 1 Laboratory of Oncovirology, Institut Pasteur du Maroc, 1 Place Louis Pasteur, 20 360 Casablanca, Morocco; 2 Laboratory of Anthropogenetics and Physiopathology of Chouaîb Doukkali University, 299 Eljadida 24 000, Morocco; 3 Biology and Medical Research Unit, Centre National de l'Energie, des Sciences et Techniques Nucléaires (CNESTEN), 10001 Rabat, Morocco; 4 Faculty of Science Ain Chock Casablanca, Morocco; 5 Service de Radiothérapie, Centre d'Oncologie IBN Rochd, Casablanca, Morocco

## Abstract

**Background:**

Nasopharyngeal carcinoma (NPC) is a malignant tumor which arises in surface epithelium of the posterior wall of the nasopharynx. There's is evidence that Epstein Barr virus (EBV) is associated to NPC development. However, many epidemiologic studies point to a connection between viral infections by the human papillomavirus (HPV) and NPC.

**Method:**

Seventy Moroccan patients with NPC were screened for EBV and HPV. EBV detection was performed by PCR amplification of BZLF1 gene, encoding the ZEBRA (Z Epstein-Barr Virus Replication Activator) protein, and HPV infection was screened by PCR amplification with subsequent typing by hybridization with specific oligonucleotides for HPV types 16, 18, 31, 33, 35, 45 and 59.

**Results:**

The age distribution of our patients revealed a bimodal pattern. Sixty two cases (88.9%) were classified as type 3 (undifferentiated carcinoma), 6 (8.6%) as type 2 (non keratinizing NPC) and only 2 (2.9%) cases were classified as type 1 (keratinizing NPC). EBV was detected in all NPC tumors, whereas HPV DNA was revealed in 34% of cases (24/70). Molecular analysis showed that 20.8% (5/24) were infected with HPV31, and the remaining were infected with other oncogenic types (i.e., HPV59, 16, 18, 33, 35 and 45). In addition, statistical analysis showed that there's no association between sex or age and HPV infection (P > 0.1).

**Conclusion:**

Our data indicated that EBV is commonly associated with NPC in Moroccan patients and show for the first time that NPC tumours from Moroccan patients harbour high risk HPV genotypes.

## Background

Nasopharyngeal carcinoma (NPC) is a tumour that arises in the epithelium surface of the posterior nasopharynx, and shows a peculiar geographic and ethnic distribution. The highest incidence rates of NPC are found among the southern Chinese population and in isolated northern populations such as Eskimos and Greenlanders (30 to 80 cases per 100,000 per year [1]. Intermediate incidence (8 to 12 cases per 100,000 per year) was reported in the Mediterranean basin, especially among the Arabic populations of North Africa (7-10% of all cancers among men), where NPC is also the commonest tumour of the ear, nose and throat region [2,3]. The etiology of NPC seems to be multifactorial with evidence that genetic, viral and other environmental factors are involved together or separately, simultaneously or consecutively [4].

Retrospectives and prospectives epidemiologic studies have indicated the association between Epstein-Barr virus (EBV), an ubiquitous human herpesvirus, and the development of different malignancies, such as Burkitt's lymphoma, 40%-50% of Hodgkin's disease, B-cell lymphoma in immunocompromised individuals, and NPC [4,5]. Undifferentiated NPC is one of the most striking examples of human malignancies that have been found strongly associated with the EBV, and interest in human papillomavirus (HPV) as a cofactor in NPC occurrences has emerged over the last few years [6].

The papillomaviruses are small double-stranded DNA viruses which infect squamous epithelia and display a very high selectivity for the specific epithelium infected [7,8]. More than 100 different HPV genotypes have been described, but only 30 genotypes identified in the female genital tract are associated with epithelial neoplasms ranging from benign common warts to malignant carcinoma of the uterine cervix [9]. It is widely reported that in addition to HPV 16 and 18, which are frequently found in association with cervical cancer (CC), HPVs 31, 33, 35, 39, 45, 51, 52, 56, 58, 59, 68, 73 and 82, while other three as probable high-risk types (types 26, 53, and 66) are also considered as carcinogenic [10].

According to their ability to transform epithelial cells, HPV genotypes are divided into low-risk and high-risk types. Low-risk types are associated with benign lesions such as warts, while infections with high-risk types progress to malignant lesions [8-10]. It has been suggested that normal human oral epithelial cells, especially nasopharyngeal cells, could be very susceptible to persistent HPV and EBV co-infections and that EBV and high-risk HPV co-infections may play an important role in the initiation of a neoplastic transformation of human oral epithelial cells [11]. To date, different degrees of associations between HPV and NPC have been described, yet no conclusive data have been obtained. Given the particular characteristics of NPC in the Moroccan population in terms of incidence, age distribution and the predominance of specific EBV strains, we hypothesize that NPC tumours from Moroccan patients harbour a specific HPV genotype. To our knowledge, this is the first study to address the question of HPV implication as a pathogenetic cofactor in NPC patients from North Africa, and the benefit of any additional knowledge that can be used for detection cannot be overemphasized.

## Results

**Figure 1:**
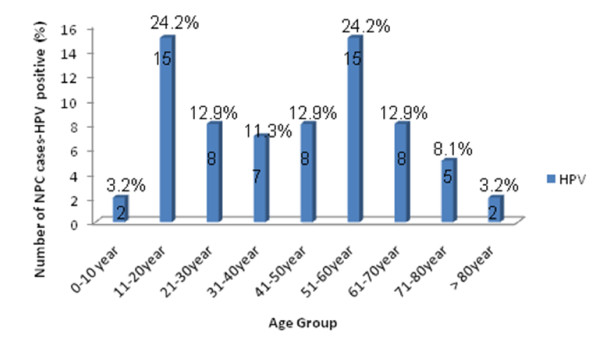
**Distribution of patients with NPC according to their age**. For each age range, number of cases and percentage are given.

The demographic characteristics of the 70 patients showed that the mean age of patients was 39.65 with extreme ages at 10 and 87 years old. The age distribution of our patients is represented in Figure 1 and revealed a bimodal pattern. The first and second peak correspond respectively to 11-20 and 51-60 years ranges. The male-to-female ratio was 2.33 to1.

The pathological analysis was performed according to the World Health Organization (WHO) classification and revealed that among the 70 cases, 62 (88.9%) were classified as type 3 (undifferentiated carcinoma), 6 (8.6%) as type 2 (non-keratinizing NPC) and only 2 (2.9%) cases were classified as type 1 (keratinizing NPC) (Table 1).

**Table 1:** Distribution of EBV and HPV infection in NPC cases

NPC cases	N	EBV+	HPV+	HPV 16	HPV 18	HPV 31	HPV 33	HPV 35	HPV 45	HPV 59	Untyped HPV cases
NPC type I	2	2	1				1				

NPC type II	6	6	1		1						

NPC type III	62	62	22	2	1	5		1	1	4	8

Total	70	70	24	2	2	5	1	1	1	4	8

N: Number of patients

The presence of amplifiable DNA, using primers for a fragment of *β-globin *gene, was confirmed for all cases and all DNA samples were adequate for further analysis. Our results show that although all NPC biopsies were EBV positive, HPV testing revealed the presence of HPV DNA in 34% of NPC cases (24/70) (Table 1).

The distribution of HPV DNA in the 24 HPV positive NPC cases is reported in (Table 1). Molecular analysis showed that HPV31 was the most common subtype and was present in 20.8% of HPV positive cases (5/24) and HPV59 in 16.7% (4/24), whereas the other high risk HPV 16, 18, 33, 35 and 45 were present in 8.3% (2/24), 8.3% (2/24), 4.2 (1/24), 4.2 (1/24) and 4.2 (1/24) respectively.

Distribution of HPV genotypes according to the anatomy pathology status showed that among the 62 NPC type III, 22 were HPV positive with a predominance of HPV31 which was present in 22.7% of cases (5/22), whereas, the only HPV-positive NPC type II biopsy was HPV-18 positive and the only HPV positive NPC type I was HPV-33 positive.

No statistically difference was observed in the HPV prevalence between males (34.7%) and females (33%) (Table 2), and no correlation between age and HPV status was found (Table 3). The mean age of HPV-positive patients was 37.3, whereas the mean age of HPV-negative cases was 43.0 years (P > 0.1).

**Table 2:** Comparison of HPV positive cases between Male and Female

Patients	N	HPV positive	P - value
Male	49	17	0.869
	
Female	21	7	

N: Number of patients

**Table 3:** Comparison of age between HPV-positive and HPV-negative cases

HPV infection	N	Mean age	P - value
HPV positive cases	24	37.3	0.425
	
HPV negative cases	46	43.0	

N: Number of patients

## Discussion

It is widely accepted that EBV is etiologically associated with NPC; but it is proven that other co-factors might be involved in the carcinogenesis process. HPVs are considered to be one of those factors since they possess the ability to transform epithelial cells and a significant number of NPC biopsies harbour HPV DNA [12,13]. We report here that 34% (24/70) from Moroccan NPC biopsies harbour HPVs. These results are in agreement with other studies reporting the same prevalence of HPV DNA in NPC cases. In fact, using the same consensus primers, HPV DNA was detected in 31 of 103 NPC samples (30%) [8]. Moreover, Krishna *et al. *have shown that HPV DNA was detected in 38.8% of 36 southern Indian NPC cases [14]. Tung *et al. *in Eighty-eight fresh tissue samples of NPC showed that HPV DNA was detected in 51% of the specimens [15].

Coinfection by HPV and EBV has not been well documented and the significance of the presence of both viruses in nasopharyngeal cells has not been determined. In our study, coinfection with both viruses was observed in 34% of patients. Tung *et al. *showed that among 88 fresh NPC specimens from Chinese population, coexistence of EBV and HPV DNA was observed in 42% of samples [15].

Of interest, EBV was detected also in CC specimens from Indonesian patients, and 68% of analysed cases are co-infected with HPV and EBV [16]. In our study, HPV and EBV co-infection seems to be less frequent in NPC. Theses differences may be due to the population' characteristics and the dissemination power of HPV in the population.

Our results show no correlation between HPV status with either age or gender in NPC patients. These findings are supported by other studies that reported no significant differences [17,18]. However, Zhang et al. showed that HPV infection either in oral squamous cell carcinoma or normal mucosa was observed with a higher frequency in men (81.3%) compared with that in women (60%) [19]. to evaluate the association between HPV status and, age and/or gender, in NPC cases, a study with a large sampling is needed.

With regard to HPV genotypes, HPV31 was the most frequent genotype in Moroccan NPC patients (20.8%). The same genotype was also frequently found in tonsils and nasopharyngeal cells in western Mexico NPC cases [20]. The second prevalent HPV type detected in our NPC biopsies is HPV59 (16,7%). Of interest, HPV-16 and -18, which are the most virulent genotypes associated with CC in Moroccan woman (35% to 45%) [21,22], were detected in very few Moroccan NPC cases (8.3%), and similar data were reported in an Iranian study [23].

Geographic and racial distinctions have been identified between NPC of the Far East versus those diagnosed in Caucasian American patients with regard to the interrelationship of histologic subtype and HPV infection. In fact, HPV are detected more often (50%) in type I NPC from American Caucasian patients than type III [6]. In our study, one of two WHO-I and 22 of 68 WHO-II/III NPCs tumors were HPV positive. The very low number of NPC type-I cases available for comparison, reflecting the rarity of WHO-I tumors in NPC patients from Morocco, did not allow us to evaluate the relationship between HPV and NPC histologic subtype.

Taken together, these data suggest that i) HPV genotypes associated with NPC are different from those consistently found in CC and ii) the HPV genotypes associated with NPC are not geo-specific.

HPV and EBV co-infections have not been well documented and the significance of the presence of both viruses in nasopharyngeal cells has not been determined. It has been shown that ZEBRA, an EBV immediate early protein expressed during lytic replication that activates early EBV genes, binds to p53 [24]. The physical interaction of the ZEBRA and p53 protein prevents p53 from activating p53-responsive promoters [25,26]. Similarly, HPV has been found to interact with p53, suggesting that this interaction promotes cell growth and thereby enhance viral replication [27]. Targeting p53 may be a common requirement for the replication of many types of DNA viruses [16]. In addition, B cells transfected with EBV latent membrane protein lost the regulatory effects of the retinoblastoma (RB) protein, and the HPV E7 transcript has been shown to immunoprecipitate the RB protein [28]. Thus, the functional loss of the RB protein might be one event common to both the HPV and EBV carcinogenic pathways. Further investigations to evaluate the expression of viral genes, especially the E6 and E7 oncogenes, are necessary to identify the possible role of HPV infection in NPC development. These investigations could lead to the development of screening programs, new therapeutic approaches and specific methods of prevention, especially in high incidence areas.

On the other hand, further studies to evaluate the impact of EBV and HPV coinfection in cervical and nasopharyngeal carcinogenesis and molecular mechanisms implicated in tumor development are warranted.

## Materials and methods

### Samples

The analyses were performed on biopsies collected from 70 patients with histopathologically confirmed NPC at the Oncology Centre in Casablanca, Morocco, after informed written consent, as approved by the local Ethical Committee. The biopsies were stored at -80°C until used.

### DNA extraction

DNA extraction was performed on frozen biopsy material. The frozen tissues were homogenized and lysed in digestion buffer (50 mM Tris-HCl, pH 8.5; 1 mM EDTA and 0.5% Tween-20) containing 200 μg ml^-1 ^of proteinase K. The samples were digested for 3 h at 55°C. Proteinase K was heat-inactivated at 95°C for 15 min. The samples were spin down and the supernatants were collected and stored at -20°C until used. In order to evaluate the efficiency of DNA extraction, all samples were polymerase chain reaction (PCR)-amplified using PC04 and GH20 primers specific for human β*-globin *gene (Table 4).

**Table 4:** Primers and specific probes used for HPV detection and typing

Primers			Sequence 5'→3'
Primers for *β-globin *amplification	PC 04		CAA CTT CAT CCA CGT TCA CC
	GH 20		GAA GAG CCA AGG ACA GGT AC

BZLF1 Primers for EBV amplification	ZES		GCC ACC TTT GCT ATC TTT GC
	ZEAS		AGG CGT GGT TTC AAT AAC GG

Primers for HPV amplification	MY 09		CGT CCM ARR GGA WAC TGA TC
	MY 11		GCM CAG GGW CAT AAY AAT GG

	HPV 16	MY14	CAT ACA CCT CCA GCA CCT AA
	HPV 18	WD74	GGA TGC TGC ACC GGC TGA
	HPV 31	WD126	CAA AAG CCC AAG GAA GAT C
Specific probes for HPV typing	HPV 33	MY16	CAC ACA AGT AAC TAG TGA CAG
	HPV 35	MY115	CTG CTG TGT CTT CTA GTG ACA G
	HPV 45	MY70	TAG TGG ACA CTA CCC GCA G
	HPV 59	MY123	GCC AGT TAA ACA GGA CCC

### PCR-quality control

To avoid contamination leading to false positive results, all PCR-related work was carried out in specialized zones within a PCR laboratory that undergoes UV purification at least once every 24 h. To detect crossover contamination negative controls (PCR reagents containing no DNA) were included in each PCR amplification. All negative controls were negative for *β-globin *and HPV assay. Positive controls containing the SiHa and Caski cell lines always amplified *β-globin *and HPV DNA, respectively.

### EBV Testing

*Z-Epstein-Barr Virus Replication Activator *(ZEBRA) gene was amplified by PCR using ZES (forward) and ZEAS (reverse) primers [29]. Primer sequences are reported in Table 4. Briefly, 2 μL of DNA were subjected to PCR in a total volume of 50 μL, which includes 5 μL of Taq DNA polymerase buffer, 50 pM of ZES/ZEAS primer mix, 0.2 μM of each dNTP (dATP, dCTP, dGTP and dTTP), and 2.5 units of Taq DNA polymerase (Promega Corp., Madison, WI). Samples were denatured at 95°C for 5 min, then cycled 30 times through 1 min denaturation at 95°C, 2 min annealing at 58°C and 2 min extension at 72°C. A final extension of 7 min at 72°C was performed. The final PCR products were analyzed by electrophoresis through a 1% agarose gel stained by ethidium bromide and optically visualized by ultraviolet transillumination.

### HPV testing

**Figure 2:**
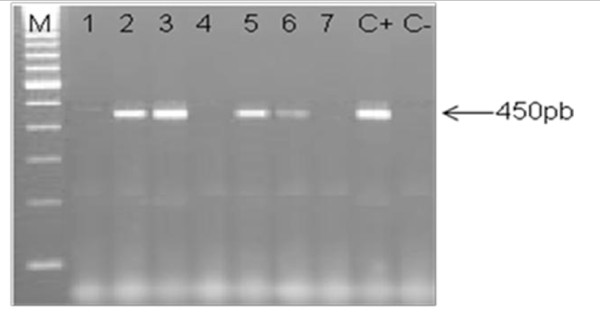
**Representative illustration of HPV detection**. Electrophoresis gel photo (A). Lanes 2, 3, 5 and 6 correspond to HPV positive NPC specimens; Lanes 1, 4 and 7 correspond to HPV negative NPC specimens; C-: negative control (sterile distilled water); C+: Positive control (HPV 31 DNA isolated from cervical cancer specimen). M: 100 bp ladder molecular weight marker.

**Figure 3:**
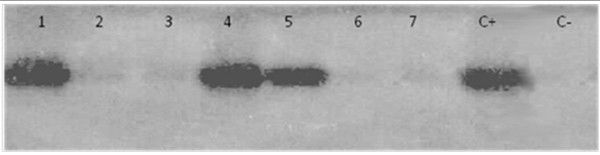
**Example of hybridization photo illustrating HPV typing**. Positive PCR products were hybridized with WD126 probe specific to HPV 31. Lanes 1, 4 and 5 correspond to HPV 31 positive cases, whereas the others (2, 3, 6 and 7) correspond to HPV 31 negative cases. C+: Positive control (HPV 31 DNA isolated from cervical cancer specimen), C-: negative control (sterile distilled water).

DNA samples were amplified by PCR using consensus primers MY09 and MY11 for a 450 bp target sequence in the L1 region that is highly conserved on a broad spectrum of HPV genotypes [29]. Each reaction mixture of 50 μl contained 50 pM of each primer, 0.2 μM of each dNTP (dATP, dCTP, dGTP and dTTP), 0.625 units of Taq DNA polymerase (Amersham Biosciences, Little Chalfont, UK), and 3 μL of DNA in Taq DNA polymerase buffer. The mixture was first denatured at 94°C for 7 min, then cycled 35 times through 30 s denaturation at 94°C, 1 min annealing at 52°C and 1 min 30 s extension at 72°C. A final extension of 7 min at 72°C was performed. Aliquots of 10 μL of the PCR product were analysed by electrophoresis through a 1.2% agarose gel. A representative gel is given in Figure 2. PCR products were transferred into positively charged nitrocellulose membranes (Hybond N+, Amersham) and fixed at 80°C for 2 h. The membranes were hybridized under stringent conditions using HPV specific biotinylated probes as described previously [30]. The probes used in this study were MY14, WD74, WD126, MY16, MY115, MY70 and MY123 specific for HPV-16, 18, 31, 33, 35, 45 and 59 respectively (Table 4) [31]. Membranes were then washed under normal and stringent conditions. Specific hybrids were detected using a biotin luminescence detection kit (Biolabs, England) and the membranes were exposed for 30 min to X-ray film (Hyperfilm ECL, Amersham). An illustrative photo is given in Figure 3.

### Statistical analysis

The results were analyzed statistically by the chi2 test. The level of significance was set at 95% (α = 0.05) for all tests.

## List of abbreviations

NPC: Nasopharyngeal carcinoma; EBV: Epstein-Barr virus; HPV: Human papillomavirus; CC: Cervical cancer; ZEBRA: Z-Epstein-Barr Virus Replication Activator; WHO: World Health Organization

## Competing interests

The authors declare that they have no competing interests.

## Authors' contributions

NL: designed experiments, contributed and analyzed data, Carried out the molecular studies and drafted the manuscript. MA: designed the primers, the approach to validation, including the selection of appropriate materials and revised the manuscript. MK: participated in study design and performed the statistical analysis. FN: participated in PCR protocol design and performed the typing test. TM: participated in PCR protocol design and performed the typing test. RD: participated in PCR protocol design and performed the typing test. KB: Participated in the conception of the study and in its coordination. NB: Collected NPC specimens and carried out the anatomy pathology analyses. MEM: designed the laboratory procedures, coordinated the laboratory quality control and revised the manuscript. MK: Conceived the study, supervised this work, participated in its design and coordination, and revised the manuscript. All authors read and approved the final manuscript.
